# NASNet-DTI: accurate drug–target interaction prediction using heterogeneous graphs and node adaptation

**DOI:** 10.1093/bib/bbaf342

**Published:** 2025-07-16

**Authors:** Ningyu Zhong, Zhihua Du

**Affiliations:** College of Computer Science and Software Engineering, Shenzhen University, No. 3688 Nanhai Avenue, Nanshan District, Shenzhen, Guangdong, 518060, China; College of Computer Science and Software Engineering, Shenzhen University, No. 3688 Nanhai Avenue, Nanshan District, Shenzhen, Guangdong, 518060, China

**Keywords:** DTI prediction, heterogeneous graph neural network, node adaptation

## Abstract

Drug–target interactions (DTIs) play a key role in drug development, and accurate prediction can significantly improve the efficiency of this process. Traditional experimental methods are reliable but time-consuming and laborious. With the rapid development of deep learning, many DTI prediction methods have emerged. However, most of these methods only focus on the intrinsic features of drugs and targets, while ignoring the relational features between them. In addition, existing graph-based DTI prediction methods often face the challenge of over-smoothing in graph neural networks (GNNs), which limits their prediction accuracy. To address these issues, we propose NASNet-DTI (Drug–target Interactions Based on Node Adaptation and Similarity Networks), a new framework designed to overcome these limitations. NASNet-DTI uses graph convolutional network to extract features from drug molecules and targets separately, and constructs heterogeneous networks to represent two types of nodes: drugs and targets. The edges in the network describe their multiple relationships: drug–drug, target–target, and drug–target. In the feature learning stage, NASNet-DTI adopts a node adaptive learning strategy to dynamically determine the optimal aggregation depth for each node. This ensures that each node can learn the most discriminative features, which effectively alleviates the over-smoothing problem and improves prediction accuracy. Experimental results show that NASNet-DTI significantly outperforms existing methods on multiple datasets, demonstrating its effectiveness and potential as a powerful tool to advance drug discovery and development.

## Introduction

Drug–target interaction (DTI) prediction plays a pivotal role in advancing diverse areas of biomedical research, including active drug discovery, polypharmacology prediction, adverse reaction forecasting, drug repositioning, drug mechanism elucidation, and protein function annotation [1–3]. Despite its critical importance, the path from initial drug development to commercial availability remains arduous, often spanning over a decade. The financial burden of this process is substantial, with total average capitalized prelaunch research and development costs varying widely, ranging from $161 million to $4.54 billion [4]. As a result, there is an urgent need for efficient methods to accelerate drug development, reduce costs, and improve the safety and efficacy of therapeutic interventions. Accurate DTI prediction offers a promising avenue to address these challenges by facilitating the rapid identification of potential drug candidates and elucidating their mechanisms of action.

Traditionally, experimental techniques such as high-throughput screening have served as the cornerstone for identifying DTIs. However, while effective, these methods are notoriously time-consuming, labor-intensive, and costly, often necessitating substantial resources and manual effort. In light of these limitations, computational approaches have emerged as a transformative alternative, enabling large-scale DTI prediction with significantly greater efficiency.

Recent advances in deep learning have further revolutionized computational DTI prediction. By leveraging vast amounts of biological data and the ability of neural networks to model intricate patterns and relationships, deep learning-based methods have demonstrated remarkable improvements in predictive accuracy and scalability. These methods can be broadly categorized into relationship network-based and structure-based approaches [5, 6].

Relationship network-based approaches like [7–10] conceptualize DTI as a graph or network problem, wherein drugs and targets are represented as nodes, and interactions are modeled as edges. These methods capitalize on the topological structure of the network, often employing graph neural networks (GNNs) or graph convolutional networks (GCNs) [11] to learn representations that encode the relationships between drugs and targets. For instance, NEDTP [12] constructs a node similarity network using 15 heterogeneous information networks. By applying random walks, it captures the topological features of each node, encodes them into low-dimensional vectors, and employs a gradient boosting decision tree (GBDT) [13] model to perform the classification task. This integration of topological information into feature learning has significantly improved predictive performance in DTI tasks.

In contrast, structure-based approaches like [14–17] focus on leveraging specific properties of drugs and targets, such as their chemical structures, sequences, and spatial configurations. These methods utilize deep learning architectures to extract patterns from these features and predict potential interactions. A representative example is HMSA-DTI [17], which processes drug Simplified Molecular Input Line Entry System (SMILES) strings, drug molecular graphs, target sequences, and target 2-mer sequences as inputs. Employing a hierarchical multimodal self-attention mechanism, HMSA-DTI effectively integrates intra- and inter-modal features of drugs and targets, enabling it to capture complex interactions and achieve superior performance in DTI prediction.

Despite these promising advances, several challenges persist. One major limitation of existing methods is the underutilization of biological knowledge graphs, which encompass rich information on molecular interactions, signaling pathways, and cellular processes. Incorporating such structured biological knowledge into DTI models has the potential to enhance their predictive power by providing a more holistic understanding of the biological context underlying DTIs. Additionally, GCN-based methods are prone to the over-smoothing problem [18], wherein node representations become indistinguishable with increasing convolutional layers. This phenomenon hampers the model’s ability to capture nuanced, fine-grained relationships between drugs and targets, thereby limiting its overall efficacy.

In this study, we introduce NASNet-DTI (**N**ode **A**daptation and **S**imilarity **Net**work-based for **D**rug-**T**arget **I**nteraction Prediction), an integrative heterogeneous graph-based framework designed for the prediction of DTIs. This framework harnesses the power of advanced GNN-based models, offering several key advantages: (i) It fully leverages the strong feature extraction capabilities of GNNs to capture comprehensive representations of both drugs and targets. (ii) It uses the extracted features to construct drug–drug edges and target–target edges, and integrates drug–target edges extracted from databases to effectively establish complex relationships between drugs and targets. This approach enriches the model’s knowledge, allowing it to capture more subtle and biologically meaningful interactions. (iii) To address the well-known issue of over-smoothing in GNNs, particularly in large graphs, we incorporate node-dependent local smoothing (NDLS) [18], a sophisticated regularization technique. NDLS dynamically adjusts the smoothing strength for each node based on its local network context, ensuring the preservation of feature uniqueness and preventing the loss of important discriminative information due to over-smoothing.

Experimental evaluations on multiple benchmark datasets demonstrate the superiority of NASNet-DTI over state-of-the-art approaches in terms of both accuracy and robustness. These findings underscore the potential of integrating biological knowledge and addressing GCN limitations to advance computational DTI prediction, offering a robust and efficient tool to accelerate drug discovery.

## Methods

### Overview of NASNet-DTI

**Figure 1:**
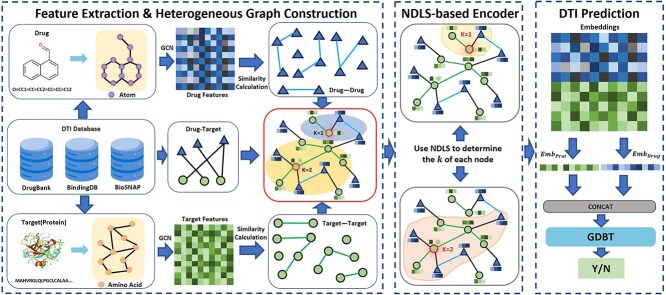
Overview of NASNet-DTI. **Feature extraction:** extraction of drug and target features and construct drug–drug edges and target–target edges; **heterogeneous graph construction:** constructing heterogeneous graphs using extracted features and edges; **NDLS-based encoder:** use the NDLS learning strategy to obtain the features of all drugs and targets; **DTI prediction:** using learned features for DTI prediction.

Our proposed method integrates multiple components to achieve robust and accurate DTI prediction. The overall framework consists of four main components: feature extraction component, heterogeneous graph construction component, NDLS-based encoder component, and the DTI prediction component, as illustrated in Fig. 1.

In the feature extraction module, we harness the powerful capabilities of GCNs to extract meaningful and representative features from drug and target nodes. These features form the foundation for constructing drug–drug edges and target–target edges, which are designed to effectively capture the underlying chemical and biological relationships between drugs and targets. In the subsequent heterogeneous graph construction module, we integrate these drug–drug edges and target–target edges with drug–target edges to form a comprehensive heterogeneous graph.

To effectively model the complex interaction patterns embedded in the heterogeneous graph, we employ the NDLS [18] algorithm in the feature encoding process. The NDLS algorithm adaptively adjusts the number of feature aggregation iterations for each node based on its local topological properties. This adaptive mechanism enables the model to retain fine-grained distinctions between nodes, addressing the over-smoothing issue prevalent in traditional GCN-based methods.

Finally, in the DTI prediction module, the encoded node features are fed into a GBDT [13] model. The GBDT model utilizes these discriminative and enriched feature representations to predict DTIs with high precision and reliability.

In the following sections, we will provide a detailed introduction to each module of the proposed method, elucidating their roles and contributions to the overall framework.

### Drugs feature extraction

To represent the chemical structure of a drug, we first utilize RDKit [19] to convert the SMILES descriptor into a molecular graph $G_{d}=(V_{d}, E_{d})$. In this molecular graph, $V_{d}$ represents the set of nodes, where each node corresponds to an individual atom, and $E_{d}$ represents the set of edges, which denote the bonding relationships between atoms.

For each atom in the molecular graph, we extract a set of features $x_{i}$ that capture its chemical properties and structural characteristics. These features include

- The atomic number $z_{i}$, which identifies the type of atom.
- The number of bonds $b_{i}$ the atom forms with neighboring atoms.
- The number of attached hydrogen atoms $h_{i}$.
- The valence state $q_{i}$, representing the atom’s bonding capacity.
- A binary indicator $c_{i}$ specifying whether the atom is part of an aromatic ring.

These features collectively encode the chemical and structural properties of each atom, enabling the model to comprehensively represent the molecular graph. Consequently, the feature vector $x_{i}$ for each atom can be mathematically expressed as shown in equation (1).


(1) \begin{eqnarray*} x_{i} = [z_{i}, b_{i}, h_{i}, q_{i}, c_{i}] \end{eqnarray*}

In addition to node features, the molecular graph also incorporates the connections between atoms through an adjacency matrix $A$. Each bond in the molecule contributes to this matrix, where $A$ encodes the connectivity between nodes (atoms) within the graph. Using this adjacency matrix, we apply a GCN to aggregate features across the molecular graph. This feature aggregation process updates the representation of each node by combining its own features with those of its neighbors, allowing the model to capture local structural information. The feature aggregation in the GCN can be described mathematically by the following equations:


(2) \begin{eqnarray*} X^{(k)}=\sigma(\bar{A}X^{(k-1)}W^{(k-1)}); \end{eqnarray*}

(3) \begin{eqnarray*} \bar{A} = D^{(r-1)}AD^{(r-1)}, \end{eqnarray*}

where $W$ is the trainable parameter matrix, $\sigma (\cdot )$ is an activation function, $X^{(k)}$ is the feature matrix after $k$ convolutions, $D=[d_{ij}]$ is the diagonal node degree matrix of graph $G$, $r$ is the convolution coefficient, and $\bar{A}$ is the adjacency matrix after symmetric normalization.

This aggregation process iteratively propagates information through the graph, enabling the model to learn enriched node embeddings that reflect the local topology and chemical environment of each atom.

To further capture the global structural and chemical information of the entire molecule, we employ average pooling as the readout function. By averaging the embeddings of all nodes within the graph, we generate a 128-dimensional vector representation for the molecule that encodes both the global structural and chemical characteristics, providing a comprehensive representation suitable for downstream tasks.

### Targets feature extraction

Let the amino acid sequence be $S=\left \{ s_{1}, s_{2},...,s_{n} \right \}$, where $s_{i}$ is the $i$th amino acid in the sequence. To enable numerical encoding of the sequence, we define an amino acid index dictionary $M$, which maps each amino acid $s_{i}$ to a unique integer index $M\left ( s_{i} \right )$. This encoding facilitates computational processing while preserving the inherent order of the sequence. Using this numerical representation, we transform the encoded amino acid sequence into a graph $G_{p}=(V_{p}, E_{p})$, where $V_{p}$ is the set of nodes, each corresponding to an amino acid, and $E_{p}$ is the set of edges, representing the connectivity between amino acids in the sequence. For each amino acid $s_{i}$ a node $v_{i}$ is created, and its feature is represented as $x_{i} = M(s_{i})$. This graph-based representation maintains the sequential information of the amino acid sequence while providing a foundation for capturing the structural and relational characteristics of the target.

To extract meaningful features from the target graph, we apply the method described in the Drug Feature Extraction section, leveraging equations (2) and (3). Using this approach, we obtain a 128-dimensional feature vector for the target. This process not only aggregates local node features to derive a global representation of the target but also offers novel perspectives and tools for understanding target structure and function. The resulting global features encapsulate both the sequential and structural properties of the target, making them highly informative for downstream tasks such as target function prediction and DTI modeling.

### Construction of drug–target heterogeneous graph

To capture more comprehensive and nuanced information between drugs and targets, we utilize the features extracted by the aforementioned method to find the correlation between drugs and targets.

In establishing drug–drug edges, we utilizes the Tanimoto coefficient [20] to quantify the chemical similarity between drug molecules, thus defining the relationships between drug–drug edges. This approach aids in identifying molecules with shared characteristics, which can be crucial in understanding potential cross-reactivity or co-action mechanisms in drug discovery [21, 22].

In establishing target–target edges, this paper employs the Smith–Waterman method [23] to detect similar target sequences in the data. From the perspective of target sequences, similarity typically suggests homology, which may imply functional similarity [24]. This helps reveal potential shared functions or effects, providing valuable insights into the biological roles of different targets and their interactions with drugs.

Finally, we extract drug–target edges from the database and integrate them with drug–drug and target–target edges to construct a heterogeneous graph $G = (V, E)$, where $V$ represents the nodes, which consist of drug and target entities, and $E$ represents the edges that connect these nodes, capturing the complex relationships between drugs and targets.

This approach enables the creation of a comprehensive and enriched heterogeneous graph that captures not only the intrinsic properties of drugs and targets but also their intricate interrelationships. By combining structural, chemical, and functional information within a unified framework, this method significantly enhances the model’s ability to accurately predict DTIs, reflecting the complex biological processes underlying these interactions.

### Joint embedding via an NDLS-based encoder

Among the methods utilizing graph-based architectures for DTI prediction, approaches such as graph attention networks [25] and GCNs [11] have exhibited notable performance due to their ability to model complex relationships in graph-structured data. These methods typically rely on a fixed propagation depth $k$, which directly affects the quality of feature aggregation and, consequently, the accuracy of the final predictions. However, selecting an optimal $k$ is nontrivial, as an insufficient depth may fail to capture long-range dependencies, while an excessive depth can lead to the well-documented issue of over-smoothing. Over-smoothing occurs when the features of nodes become indistinguishable as the propagation depth increases, ultimately impairing the model’s ability to differentiate between nodes.

To overcome this limitation, Zhang *et al*. [18] introduced NDLS, an innovative technique that addresses the drawbacks of fixed-depth propagation. By introducing the concept of Local Smoothing Iterations (LSIs) [18], NDLS computes a tailored number of propagation steps for each node, enabling adaptive smoothing that balances feature aggregation and preservation. This adaptive approach not only mitigates the over-smoothing problem but also enhances the expressiveness and robustness of learned node representations, ultimately improving predictive performance in DTI tasks.

**Figure 2:**
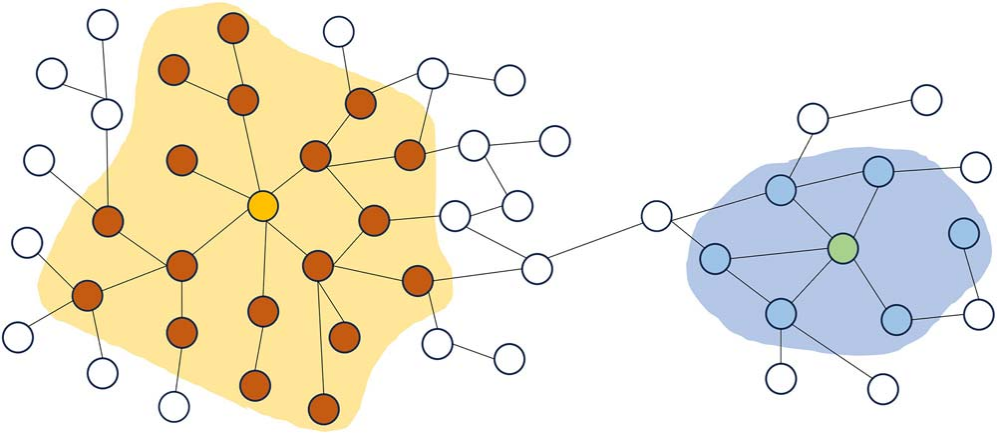
Illustration of the core idea of the NDLS algorithm.

The core idea of NDLS is that the local graph structure of different nodes and their positions in the global network are different, so the optimal number of iterative aggregations required for each node should also be different. The algorithm overcomes the defect of the traditional GCN fixed propagation depth by dynamically adjusting the number of aggregations for each node. The schematic diagram of the NDLS algorithm is shown in Fig. 2. The calculated optimal number of iterative aggregations required for the yellow and green nodes in the figure is 2 and 1, respectively.

The algorithm process of NDLS will be introduced in detail below:

In GCN, node features are updated iteratively by aggregating information from their neighboring nodes. After $k$ iterations of propagation, the features of the nodes can be expressed as $X^{(k)}=\hat{A}^{k}X$, where $\hat{A}$ is the normalized adjacency matrix that encodes the graph structure, and $X$ is the initial feature matrix. This iterative process facilitates the integration of information from progressively larger neighborhoods, capturing both local and global graph characteristics.

To quantify the extent to which node $V_{j}$ influences node $V_{i}$ after $k$ iterations, an influence matrix $I_{h}(k)$ is defined. For the $h$th feature of the feature matrix $X$, the element $I_{h}(k)_{ij}$ of the influence matrix is expressed as


(4) \begin{eqnarray*} I_{h}(k)_{ij}=\frac{\partial \hat{X}^{(k)}_{ih}}{\partial \hat{X}^{(0)}_{ih}}, \end{eqnarray*}

where $\hat{X}^{(k)}_{ih}$ denotes the $h$th feature of node $v_{i}$ after the $k$th iteration, and $\hat{X}^{(0)}_{ih}$ represents the original $h$th feature of node $V_{j}$. The influence vector for any node $v_{i}$ thereby captures the cumulative effect of all other nodes in the graph on $v_{i}$ after $k$ iterations. As the number of propagation steps increases, the features of each node are influenced not only by its immediate neighbors but also by nodes located farther away within the graph. This property underpins the ability of GCNs to aggregate information across long-range connections, providing a comprehensive representation of the graph’s structure and node-level attributes.

Zhang *et al*. [18] introduced the concept of LSI to quantify the minimum number of iterations $k$ required for the features of a node $v_{i}$ to achieve over-smoothing stability within a predefined tolerance $\varepsilon $. This approach allows for an adaptive determination of propagation depth for each node, tailored to the specific structural and feature characteristics of the graph. The formal definition of LSI is as follows:


(5) \begin{eqnarray*} K(i, \varepsilon) = \min \left \{ k:\left \| \tilde{I_{i}}-I_{i}^{(k)} \right \|_{2} < \varepsilon \right \}, \end{eqnarray*}

where $\varepsilon $ represents a threshold that governs the degree of tolerance for the smoothing effect, effectively controlling how closely the node features must approximate their over-smoothing stability. $\tilde{I_{i}}$ denotes the stable state of the node $v_{i}$ under over-smoothing conditions, and $I_{i}^{(k)}$ represents the influence vector of the node after the $k$th iteration. It ensures that each node $v_{i}$ undergoes the optimal number of smoothing iterations necessary to preserve meaningful distinctions in its features while avoiding excessive uniformity. By defining LSI in this manner, the method addresses the limitations of fixed propagation depths in traditional GCN architectures.

Once the minimal value of $k$ for learning $X_{i}$ is determined, an averaging operation is applied to aggregate sufficient neighborhood information within $k$-hops from node $v_{i}$. The updated representation of $X_{i}$ is then computed using the following update rule:


(6) \begin{eqnarray*} X_{i} = \frac{1}{K(v_{i},\varepsilon )+1}\sum_{k=0}^{K(v_{i},\varepsilon )}X_{i}^{(k)} \end{eqnarray*}

By tailoring the extent of feature propagation to each node’s local graph structure and characteristics, the model effectively avoids the detrimental effects of over-smoothing. Consequently, this approach enhances the representational power of the node embeddings and improves the overall performance of the graph-based learning task.

### DTI prediction via GBDTs

GBDTs [13] is a powerful ensemble learning algorithm widely used for both classification and regression tasks. GBDT improves predictive performance by iteratively constructing decision trees in a stage-wise manner to minimize a specified loss function. The algorithm employs a process known as ”boosting,” where each successive tree is trained to correct the residual errors of the previous trees. This incremental approach enables the model to focus more on samples with larger prediction errors, thereby refining its overall accuracy.

Following the above steps, we derive the embedding vector matrix $X$ for all drug and target nodes in the heterogeneous graph.

For a given drug–target pair $<v_{i}, v_{j}>$, we extract their respective embedding vectors, $X_{i}$ and $X_{j}$ from the matrix $X$. These embeddings are concatenated to form a joint feature vector, $emb_{k} = [X_{i}, X_{j}]$. Then, we combine all the joint features in the matrix $\mathbf{EMB} = [emb_{1}, emb_{2},..., emb_{l}]$, which serves as the input to the GBDT classifier [13].

Furthermore, we define a prediction result vector $\mathbf{R} = [R_{1}, R_{2},..., R_{m}]$, where $m$ denotes the size of the test set. Each element $R_{k}$ in $\mathbf{R}$ represents the predicted likelihood of interaction between the respective drug and target nodes. Higher scores indicate a stronger likelihood of interaction, providing a quantifiable measure for predicting potential DTIs. The complete pseudo-code of the proposed algorithm is presented in Algorithm 1.



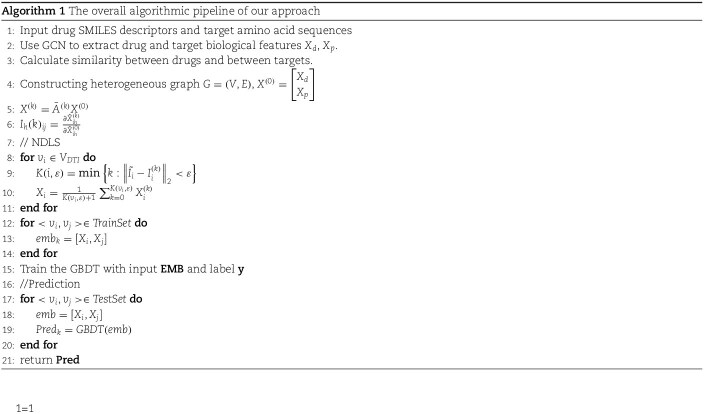



## Results and discussion

### Benchmark datasets

We evaluate NASNet-DTI using three widely recognized datasets: DrugBank [26], BioSNAP [27], and BindingDB [28], comparing their performance with five baseline models: Random Forest (RF) [29], GBDT [13], DrugBAN [16], iGRLDTI [7], and HMSA-DTI [17]. Furthermore, to assess the robustness of our approach under real-world conditions, we construct an imbalanced version of each dataset, where the ratio of positive to negative samples is $\sim $1:10. To ensure robustness and reliability, all our experiments were conducted using 10-fold cross-validation. A detailed description of these datasets is provided in Table 1. Below are brief descriptions of each dataset.

**Table 1:** A detailed description of datasets

Dataset	Drugs	Targets	Positive	Negative
DrugBank	6645	4254	17 511	17 511
BioSNAP	14 643	2623	20 674	28 525
BindingDB	4505	2181	13 830	13 634

Alt Text: Tabular summary of the three benchmark datasets (DrugBank, BioSNAP, BindingDB), showing the number of drugs, targets, and positive/negative samples in each dataset.

#### DrugBank dataset

DrugBank [26] is a comprehensive and freely accessible web resource that provides detailed information on drugs, their targets, mechanisms of action, and response profiles. It encompasses a wide range of data on both FDA-approved drugs and experimental drugs undergoing the FDA approval process. The resource is renowned for its rich, high-quality, and original source content, particularly the detailed sequences associated with drugs and their targets. These attributes have established DrugBank as one of the most widely utilized reference resources in the field of drug research globally. Its extensive database serves a broad audience, including the general public, educators, pharmacists, pharmacologists, medicinal chemists, drug researchers, and professionals, in the pharmaceutical industry [30].

In our study, we utilized the DrugBank Dataset obtained from previous work [17], which comprises a dataset of 6645 drugs and 4254 targets, resulting in a total of 17 511 drug–target edges and 17 511 negative samples.

#### BioSNAP dataset

BioSNAP [27] is a comprehensive collection of biomedical network datasets provided by Stanford University. It includes a variety of datasets that represent different types of biomedical relationships, such as drug–drug interactions, drug–gene interactions, and target–target interactions. Due to its wide range and high-quality data, BioSNAP is frequently used in bioinformatics and pharmacology research, particularly in DTI prediction. The BioSNAP dataset we utilized in this study is obtained from previous work [16], which consists of 14 643 drugs and 2623 targets, with a total of 20 674 drug–target edges and 28 525 negative samples.

#### BindingDB dataset

BindingDB [28] is a publicly accessible, web-based database that compiles extensive affinity data between drug-like small molecules and targets identified as potential drug targets. Developed and maintained by Michael K. Gilson’s laboratory at the University of California, San Diego, the BindingDB dataset we utilized in this study is obtained from previous work [16], which consists of 4505 drugs and 2181 targets, with a total of 13 830 DTI edges and 13 634 negative samples.

### Baseline methods

#### Random forest

RF [29] is an ensemble learning algorithm that constructs multiple decision trees for classification or regression tasks. Introduced by Leo Breiman and Adele Cutler in 2001, RF has gained widespread recognition for its robust prediction performance and its capability to manage high-dimensional data efficiently. The key principle of RF lies in aggregating predictions from multiple decision trees, thereby enhancing the overall accuracy and robustness of the model.

In our study, we employ RF as a benchmark model to evaluate the effectiveness of our proposed Drug Feature Extraction and Target Feature Extraction methods. By directly using the extracted features as input, we aim to demonstrate the ability of our feature representations to support accurate DTI predictions.

#### Gradient boosting decision tree

GBDT, as previously introduced, is not discussed again here.

Like RF, we only use the features extracted in Drug Feature Extraction and Target Feature Extraction for prediction to verify the effectiveness of our feature extraction method.

#### DrugBAN

DrugBAN [16] is a deep bilinear attention network framework tailored for DTI prediction. It integrates domain adaptation techniques to explicitly model pairwise local interactions between drugs and targets while adapting to out-of-distribution data. DrugBAN processes drug molecular graphs and target sequences as inputs, leveraging conditional domain adversarial learning to align the learned interaction representations across different distributions, thereby enhancing the generalization capability for novel drug–target edges. One of the distinctive features of DrugBAN is its bilinear attention mechanism, which enables the model to capture fine-grained interactions between drugs and targets. Furthermore, by visualizing the bilinear attention map, DrugBAN provides an added layer of explainability, offering insights into the underlying interaction patterns that contribute to the prediction results.

#### iGRLDTI

iGRLDTI [7] aims to predict DTI by constructing a heterogeneous network that only integrates DTIs. However, this method does not fully leverage the relationships between drugs or between targets, nor does it incorporate the structural characteristics of targets during feature extraction.

#### HMSA-DTI

HMSA-DTI [17] is a structure-based hierarchical multimodal self-attention GNN designed for DTI prediction. It takes as input drug SMILES strings, drug molecular graphs, target sequences, and target 2-mer sequences. By employing a hierarchical multimodal self-attention mechanism, HMSA-DTI achieves deep fusion of multimodal features for drugs and targets. This enables the model to effectively capture both internal and cross-modal interactions between drugs and targets, leading to improved prediction accuracy.

### Performance comparison on benchmark datasets

This section presents a detailed comparison of NASNet-DTI’s performance against the other models. We employ Area Under Curve (AUC), Area Under the Precision-Recall Curve (AUPR), F1 score, Sensitivity (Recall), and Specificity as performance evaluation metrics. The calculation formula is as follows:


(7) \begin{eqnarray*} F1 = 2 \times \frac{Precision \times Recall}{Precision + Recall}; \end{eqnarray*}

(8) \begin{eqnarray*} Precision = \frac{TP}{TP+FN}; \end{eqnarray*}

(9) \begin{eqnarray*} Sensitivity(Recall) = \frac{TP}{TP+FN}; \end{eqnarray*}

(10) \begin{eqnarray*} Specificity = \frac{TN}{TN+FP}, \end{eqnarray*}

where $TN$ represents the number of true negative examples and $FP$ represents the number of false positive examples.

AUC and AUPR offer an overarching assessment of the model’s performance across all possible thresholds. The F1 score, which combines precision and recall, provides a balanced measure of the effectiveness of the model. Sensitivity and specificity evaluate the model’s capability to correctly classify positive and negative instances, respectively. The comprehensive results are summarized in Table 2.

**Table 2:** Comparison of NASNet-DTI and the baseline model on three different datasets using random splits.(RF: Random Forest, GBDT: Gradient Boosted Decision Tree. These two models use only the drug and target features extracted through the aforementioned method, concatenated as input)

**Dataset**	**Method**	**AUC**	**AUPR**	**F1**	**Sensitivity**	**Specificity**
DrugBank	RF	0.820 $\pm $ 0.0038	0.839 $\pm $ 0.0031	0.742 $\pm $ 0.0035	0.666 $\pm $ 0.0044	0.851 $\pm $ 0.0047
	GBDT	0.834 $\pm $ 0.0051	0.854 $\pm $ 0.0033	0.762 $\pm $ 0.0057	0.722 $\pm $ 0.0069	0.828 $\pm $ 0.0059
	DrugBAN	0.856 $\pm $ 0.0061	0.859 $\pm $ 0.0055	0.786 $\pm $ 0.0070	0.757 $\pm $ 0.0098	0.822 $\pm $ 0.0077
	iGRLDTI	0.918 $\pm $ 0.0041	0.914 $\pm $ 0.0048	**0.860 $\pm $ 0.0077**	0.834 $\pm $ 0.0112	0.853 $\pm $ 0.0090
	HMSA-DTI	0.922 $\pm $ 0.0053	0.919 $\pm $ 0.0049	0.833 $\pm $ 0.0073	**0.856 $\pm $ 0.0088**	0.826 $\pm $ 0.0073
	**NASNet-DTI**	**0.934 $\pm $ 0.0042**	**0.932 $\pm $ 0.0044**	0.846 $\pm $ 0.0071	0.848 $\pm $ 0.0106	**0.869 $\pm $ 0.0087**
BioSNAP	RF	0.903 $\pm $ 0.0038	0.904 $\pm $ 0.0039	0.837 $\pm $ 0.0049	0.828 $\pm $ 0.0051	0.849 $\pm $ 0.0066
	GBDT	0.865 $\pm $ 0.0041	0.877 $\pm $ 0.0033	0.789 $\pm $ 0.0043	0.754 $\pm $ 0.0055	0.842 $\pm $ 0.0061
	DrugBAN	0.905 $\pm $ 0.0049	0.905 $\pm $ 0.0048	0.845 $\pm $ 0.0055	0.830 $\pm $ 0.0059	0.856 $\pm $ 0.0065
	iGRLDTI	0.924 $\pm $ 0.0036	0.927 $\pm $ 0.0039	0.841 $\pm $ 0.0056	0.829 $\pm $ 0.0072	0.868 $\pm $ 0.0078
	HMSA-DTI	0.930 $\pm $ 0.0044	0.937 $\pm $ 0.0045	0.863 $\pm $ 0.0050	0.820 $\pm $ 0.0067	0.847 $\pm $ 0.0062
	**NASNet-DTI**	**0.942 $\pm $ 0.0038**	**0.941 $\pm $ 0.0041**	**0.864 $\pm $ 0.0059**	**0.845 $\pm $ 0.0077**	**0.882 $\pm $ 0.0073**
BindingDB	RF	0.835 $\pm $ 0.0035	0.857 $\pm $ 0.0040	0.758 $\pm $ 0.0041	0.697 $\pm $ 0.0050	0.858 $\pm $ 0.0049
	GBDT	0.906 $\pm $ 0.0031	0.905 $\pm $ 0.0041	0.833 $\pm $ 0.0045	0.815 $\pm $ 0.0055	0.858 $\pm $ 0.0050
	DrugBAN	0.960 $\pm $ 0.0051	0.948 $\pm $ 0.0049	0.900 $\pm $ 0.0053	0.897 $\pm $ 0.0063	0.907 $\pm $ 0.0066
	iGRLDTI	0.932 $\pm $ 0.0043	0.933 $\pm $ 0.0044	0.853 $\pm $ 0.0048	0.855 $\pm $ 0.0053	0.859 $\pm $ 0.0059
	HMSA-DTI	0.974 $\pm $ 0.0040	0.924 $\pm $ 0.0045	**0.962 $\pm $ 0.0042**	0.900 $\pm $ 0.0057	0.908 $\pm $ 0.0055
	**NASNet-DTI**	**0.982 $\pm $ 0.0034**	**0.981 $\pm $ 0.0038**	0.926 $\pm $ 0.0046	**0.913 $\pm $ 0.0061**	**0.934 $\pm $ 0.0065**

Alt Text: Performance comparison of NASNet-DTI and five baseline models (RF, GBDT, DrugBAN, iGRLDTI, HMSA-DTI) on three datasets, using metrics such as AUC, AUPR, F1, Sensitivity, and Specificity. NASNet-DTI achieves top AUC and AUPR across all datasets.

In our study, we set out to evaluate the effectiveness of our feature extraction technique by applying it to two established baseline methods: RF and GBDT. The integration of our feature extraction method led to both baseline models demonstrating commendable AUC and AUPR scores. Notably, on the BioSNAP dataset, RF’s performance was strikingly close to that of the sophisticated DrugBAN model, indicating that our feature extraction approach is highly effective in capturing critical aspects of DTIs.

Experimental results on three benchmark datasets demonstrate that NASNet-DTI achieves competitive performance in predicting DTIs. On the DrugBank dataset, NASNet-DTI obtained the highest AUC (0.934) and AUPR (0.932), while HMSA-DTI showed the best sensitivity (0.856), and iGRLDTI achieved the best F1 score (0.860). This suggests that while NASNet-DTI performs strongly overall, especially in ranking-based metrics, no single model consistently dominates across all evaluation criteria.

Similar observations can be made on the BioSNAP and BindingDB datasets. NASNet-DTI achieved the highest AUC and specificity on all three datasets, and also performed competitively in other metrics such as AUPR and F1. However, other models, such as HMSA-DTI, also performed strongly in metrics like sensitivity and F1 score, reflecting their strengths in different aspects of DTI prediction.

These varying performances may be attributed to the differences in model architecture and their alignment with data characteristics. For instance, HMSA-DTI incorporates a hierarchical multimodal self-attention mechanism to fuse local structural and sequential features of drugs and targets. This design may be particularly advantageous in detecting interactions determined by fine-grained chemical or sequence motifs, leading to higher sensitivity and recall. In contrast, NASNet-DTI utilizes an NDLS-based GNN over a heterogeneous biological information network, emphasizing relational and functional associations between drugs and targets. This may explain its superior performance on global evaluation metrics such as AUC and AUPR.

These results indicate that no model is universally optimal across all criteria. Instead, each model exhibits specific strengths, and the choice of model may depend on the particular application requirements or characteristics of the dataset.

### Comparison of simulated real-world imbalanced data

In order to simulate the situation in the real world where positive samples are far less than negative samples, we constructed three imbalanced datasets with a positive-to-negative sample ratio of 1:10 for experimental evaluation. Experimental results are shown in Table 3.

**Table 3:** Comparison of NASNet-DTI and the baseline model on imbalance datasets(Positive:Negative = 1:10)

Dataset	Method	AUC	AUPR	F1
DrugBank(Imbalance)	DrugBAN	0.898	0.682	**0.872**
	iGRLDTI	0.899	0.682	0.593
	HMSA-DTI	0.824	0.534	0.709
	**NASNet-DTI**	**0.942**	**0.714**	0.719
Bindingdb(Imbalance)	DrugBAN	0.962	0.861	**0.912**
	iGRLDTI	0.976	0.877	0.771
	HMSA-DTI	0.921	0.729	0.846
	**NASNet-DTI**	**0.979**	**0.883**	0.804
BioSNAP(Imbalance)	DrugBAN	0.923	0.723	0.856
	iGRLDTI	0.936	0.702	0.603
	HMSA-DTI	0.938	**0.789**	**0.858**
	**NASNet-DTI**	**0.942**	0.754	0.702

Alt Text: Model performance on artificially imbalanced datasets with a 1:10 positive-to-negative ratio. NASNet-DTI outperforms others in AUC and AUPR, maintaining high prediction capability under class imbalance.

Across all three datasets, NASNet-DTI consistently demonstrated significant improvements in both AUC and AUPR, which are key metrics for assessing classifier performance in imbalanced settings. The AUC values for NASNet-DTI were 0.942, 0.979, and 0.942 on the three imbalanced datasets, respectively. These scores represent the highest AUC achieved among all models tested, underscoring NASNet-DTI’s superior capacity to distinguish between classes across a range of thresholds. The AUPR values for NASNet-DTI were 0.714, 0.883, and 0.754 on the respective datasets. These results are particularly significant as they highlight NASNet-DTI’s enhanced capability to identify positive samples within an imbalanced dataset. For instance, on the BindingDB (imbalance) dataset, NASNet-DTI’s AUPR of 0.883 surpasses the next best model (iGRLDTI at 0.877), demonstrating its effectiveness in maintaining high precision and recall for the positive class.

In addition, we also report the F1-score to provide a more comprehensive evaluation. Despite the inherent difficulty of achieving high F1-scores under extreme class imbalance, NASNet-DTI still achieved competitive results, with F1-scores of 0.719, 0.804, and 0.702 on the DrugBank, BindingDB, and BIOSNAP datasets, respectively. These results reflect the model’s capacity to maintain a reasonable balance between precision and recall, even when the number of positive samples is limited.

The performance of NASNet-DTI on imbalanced datasets has important implications for the field of bioinformatics. In the context of DTI prediction, accurately identifying positive interactions is crucial for drug discovery and repurposing. The high AUC, AUPR, and competitive F1-scores achieved by NASNet-DTI highlight its robustness and generalizability under real-world data distributions, paving the way for more reliable predictions and ultimately a more efficient drug development process.

### Ablation study

#### Effect of different feature embedding dimension on prediction performance

To investigate the impact of embedding vector dimensionality on model performance, we conducted an ablation study using five different embedding dimensions (32, 64, 128, 256, and 512) on the DrugBank dataset with NASNet-DTI. As shown in the Table 4, the 128-dimensional embeddings consistently yielded the highest performance across all evaluation metrics.

**Table 4:** Performance of different feature dimensions on the DrugBank dataset

	AUC	AUPR	F1	Sensitivity	Specificity
32dim	0.912	0.910	0.820	0.818	0.838
64dim	0.920	0.928	0.838	0.822	0.853
**128dim**	**0.934**	**0.932**	**0.846**	**0.848**	**0.869**
256dim	0.897	0.903	0.810	0.787	0.842
512dim	0.847	0.864	0.770	0.734	0.828

Alt Text: Evaluation of NASNet-DTI using different embedding sizes (32, 64, 128, 256, 512) on the DrugBank dataset. The 128-dimensional embedding consistently yields the best performance across AUC, AUPR, F1, Sensitivity, and Specificity.

These results suggest that 128-dimensional embeddings strike a favorable balance between representational capacity and model complexity. On the one hand, lower dimensional embeddings (e.g. 32 and 64) may lack sufficient expressive power to capture the complex semantic relationships inherent in DTIs, resulting in suboptimal performance. On the other hand, higher dimensional embeddings (e.g. 256 and 512) tend to lead to performance degradation, potentially due to overfitting caused by increased model capacity and noise sensitivity. This observation highlights the importance of selecting an appropriate embedding dimensionality to optimize model generalizability and predictive accuracy.

#### Effect of additional biological information on prediction performance

**Figure 3:**
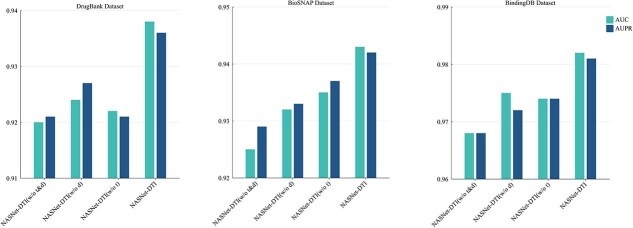
Ablation experiments on three datasets to investigate the effect of adding different biological information graphs on prediction performance.

Integration of biological information plays a pivotal role in enhancing the predictive accuracy of models. To assess the contribution of incorporating target–target and drug–drug relationships into the heterogeneous graph construction for DTI prediction, we conducted an ablation experiment. This experiment evaluated four model variants: **NASNet-DTI ($w/o\ t\&d$)**, which excludes both target–target and drug–drug edges; **NASNet-DTI ($w/o\ d$)**, which excludes drug–drug relationships; **NASNet-DTI ($w/o\ t$)**, which excludes target–target relationships; and **NASNet-DTI**, the full model that integrates both target–target and drug–drug relationships. These variations allowed for a systematic investigation of how these edges contribute to the model’s ability to capture meaningful biological relationships and improve predictive performance. Experimental results are shown in Fig. 3.

Experimental results demonstrate that the complete model, NASNet-DTI, outperforms all other models across all datasets. For instance, on the DrugBank dataset, NASNet-DTI achieves an AUC of 0.934 and an AUPR of 0.932, significantly outperforming models that only incorporate a single type of edge pair. For example, NASNet-DTI ($w/o\ d$) achieves an AUC of 0.924 and an AUPR of 0.927, while NASNet-DTI ($w/o\ p$) has an AUC of 0.922 and an AUPR of 0.921. In comparison, NASNet-DTI ($w/o\ t\&d$), which excludes both target–target and drug–drug edges, shows an AUC of 0.920 and an AUPR of 0.921. These findings are consistent across other datasets, validating that integrating target–target and drug–drug edges significantly enhances the model’s ability to capture the complex interactions between drugs and targets, thereby improving prediction accuracy.

The performance improvement observed in NASNet-DTI can be attributed to the integration of biological relevance into the model. Target–target edges capture the evolutionary and functional relationships between targets, offering insights into potential interactions with drugs. Drug–drug edges, on the other hand, reflect structural and pharmacological similarities between drugs, which can help identify potential cross-reactions or co-action mechanisms. By incorporating these biological and chemical insights, NASNet-DTI is able to make more precise and biologically meaningful predictions, advancing the accuracy and reliability of DTI prediction.

#### The impact of learning strategies on prediction performance

**Figure 4:**
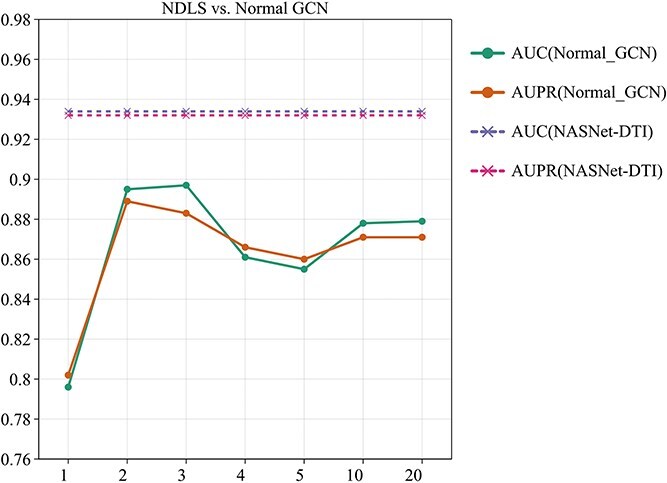
Ablation experiments on DrugBank to investigate the effect of NDLS and normal GCNs on prediction performance. (The x-axis represents the number of layers of GCN.)

To evaluate the impact of the node-adaptive feature learning strategy on DTI prediction, we conducted an ablation experiment using the DrugBank dataset. The experiment systematically compared the performance of the NDLS method with that of the standard GCN model, which contained 1–20 layers. By comparing these two approaches, we aim to better understand the role of adaptive node propagation in improving the prediction accuracy of DTIs and overcoming challenges such as over-smoothing typically encountered in deep GCN models. The detailed results are illustrated in Fig. 4.

Our NDLS-based method demonstrated a remarkable performance, achieving the highest AUC of 0.934 and AUPR of 0.932. These results highlight its superior ability to effectively extract and utilize graph-structured data features, a critical requirement for accurate DTI prediction.

In contrast, the standard GCN models exhibited a non-monotonic performance trend across different layer configurations. Initially, increasing the number of layers from 1 to 2 significantly improved performance, with AUC rising from 0.796 to 0.895 and AUPR increasing from 0.802 to 0.889. This suggests that additional layers enhance the model’s capacity to capture more complex and meaningful features from the data.

However, as the layer count increased to 4 and 5, performance began to decline, with AUC dropping to 0.861 and 0.855, and AUPR decreasing to 0.866 and 0.860. This degradation can be attributed to the well-documented issue of over-smoothing in deeper GCNs, where node embeddings become excessively similar, eroding their discriminative power and negatively impacting prediction accuracy.

Interestingly, when the number of layers reached 10, performance exhibited a slight rebound, with AUC improving to 0.878 and AUPR to 0.871. This resurgence may reflect the model’s ability to capture higher order and more abstract features at greater depths, although it still fell short of the performance achieved by our NDLS-based approach.

Finally, further increasing the layer count to 20 resulted in only marginal gains, with AUC at 0.879 and AUPR remaining at 0.871. Despite these slight improvements, the performance of the deeper GCN models remained below that of NASNet-DTI. This underscores the efficacy of NDLS in overcoming the limitations of standard GCN architectures, such as over-smoothing, while enabling more accurate and robust predictions. These findings validate the importance of our approach in enhancing the capacity of graph-based learning for biological data applications.

### Case study

To evaluate the capability of the proposed method in identifying novel DTIs, a case study was conducted following a structured process. First, DTI data from DrugBank version 3.0 were selected as the training set, ensuring the model could learn existing DTI features effectively. Next, the trained model was applied to predict all drug–target edges not included in the training set, while excluding edges labeled as negative samples to eliminate potential training biases. Finally, the top 15 drug–target edges with the highest prediction scores were selected, and their validity was verified using the latest DrugBank version 5.1.13. This approach allowed for an accurate assessment of the model’s ability to discover novel DTIs. The results are shown in the Table 5.

**Table 5:** Top 15 drug–target edges with the highest prediction scores

Drug ID	Target ID	Evidence
DB01159	P18505	DrugBank 5.1.13
DB00255	P03372	DrugBank 5.1.13
DB00909	P21397	DrugBank 5.1.13
DB01136	P35368	DrugBank 5.1.13
DB01268	P17948	DrugBank 5.1.13
DB01520	O60391	DrugBank 5.1.13
DB00398	Q08345	Unknown
DB00909	P00918	DrugBank 5.1.13
DB01159	O60391	DrugBank 5.1.13
DB00907	Q01959	DrugBank 5.1.13
DB00677	P22303	DrugBank 5.1.13
DB00449	P25100	Unknown
DB00909	O43570	DrugBank 5.1.13
DB00594	P19801	DrugBank 5.1.13
DB02010	O14920	DrugBank 5.1.13

Alt Text: List of the top 15 predicted DTI pairs by NASNet-DTI, along with evidence from DrugBank 5.1.13 validating most predictions as true interactions, demonstrating the model’s real-world applicability.

The results of the case study revealed that 13 out of the 15 predicted drug–target edges with the highest scores were confirmed in DrugBank version 5.1.13, demonstrating the high reliability of the proposed method in predicting previously unknown DTIs.

These findings highlight the significant potential of the proposed method in advancing drug discovery and biomedical research. By accurately predicting novel DTIs, the method can accelerate the drug development process and provide valuable insights for drug repurposing. Furthermore, the method’s high accuracy and robustness offer a strong foundation for future research, supporting the development of bioinformatics and computational drug design. Its application could play a pivotal role in expediting the discovery of effective treatments and enhancing our understanding of drug–target relationships.

## Conclusion

The NASNet-DTI model introduced in this study represents a significant advancement in DTI prediction by leveraging the power of heterogeneous networks and adaptive node propagation techniques. By integrating drug molecular graphs and target structure graphs, the model constructs drug–drug relationships and protein-protein relationships that capture the complex interactions between drugs and targets while effectively learning their relational features. A key innovation of NASNet-DTI is the use of NDLS, which dynamically adjusts the aggregation depth for each node, addressing the oversmoothing problem commonly encountered in GNNs.

The results of our analysis highlight several critical findings. First, the integration of both drug–drug and target–target relationships into a heterogeneous graph enhances the model’s ability to identify complex interactions, leading to superior predictive performance. This underscores the importance of incorporating biologically relevant information into the model for accurate DTI prediction. Additionally, the NDLS strategy proves to be highly effective in overcoming the limitations of standard GCN architectures by mitigating oversmoothing and enabling more nuanced and discriminative node representations. This innovation plays a central role in the model’s enhanced accuracy and reliability.

Furthermore, our results show that the NASNet-DTI model performs well in most cases, not only on balanced datasets, but also on imbalanced ones. This robustness is crucial for real-world applications, where data often suffer from imbalance. It highlights the practical utility of NASNet-DTI in addressing the challenges of drug discovery and predicting DTIs with high reliability.

While NASNet-DTI demonstrates strong potential in DTI prediction, several limitations warrant attention. First, the current heterogeneous network incorporates only drug–drug and target–target relationships based on molecular and structural similarity, omitting other biologically informative associations such as drug–gene interactions, pathway participation, and side-effect profiles. Including these additional modalities could provide a more comprehensive biological context and enhance prediction accuracy. Second, the model is limited to binary classification and does not characterize interaction details such as binding affinity, kinetics, mechanisms of action (e.g. agonist or antagonist), or specific binding sites. Such information is critical for practical applications in drug development and mechanistic understanding. Third, the model lacks explicit strategies to handle noisy data. One notable source of noise stems from the generation of negative samples: many unknown drug–target pairs are treated as negatives, although some may in fact represent undiscovered positives. This issue introduces potential label noise and remains a challenge in the DTI prediction field.

Future work could aim to address these limitations by incorporating richer biological data, refining task formulations to capture interaction properties, and exploring advanced learning paradigms to mitigate label noise and improve model generalization.

**Key Points**

- Incorporating both drug–drug and target–target edges into the heterogeneous graph greatly enhances the model’s ability to capture intricate interactions, leading to superior predictive performance. This underscores the biological relevance of these networks in accurate DTI prediction.
- The NDLS strategy successfully addresses the oversmoothing problem inherent in standard GCN architectures. By enabling adaptive node propagation, NDLS allows for more nuanced and discriminative feature representation, translating into marked improvements in prediction accuracy.
- Comparative analysis with state-of-the-art methods, including DrugBAN, iGRLDTI, and HMSA-DTI, demonstrates that NASNet-DTI consistently outperforms these models, particularly in imbalanced datasets where positive interactions are underrepresented. This robustness is critical for real-world applications.

## Data Availability

The code and data are available at https://github.com/Bo-Zhong-00/NASNet-DTI
